# Calcium and potassium silicates impact life table parameters of *Tuta absoluta* (Lepidoptera: Gelechiidae) and improve field performance of tomato (*Solanum lycopersicum*)

**DOI:** 10.1038/s41598-026-51521-y

**Published:** 2026-05-09

**Authors:** Samaneh Akbari, Shahram Aramideh, Mahdieh Mousavi

**Affiliations:** 1 Department of Plant Protection, Faculty of Agriculture, Urmia University, Urmia, Iran; 2 Plant Protection Research Department, Agricultural and Natural Resources Research and Education Center, Kurdistan Province, Agricultural Research, Education and Extension Organization, Sanandaj, Iran

**Keywords:** Tomato fruit yield, *Tuta absoluta*, Leaf-miner damage, Postharvest quality, Weight loss, Decay rate, Integrated pest management, Ecology, Ecology, Physiology, Plant sciences, Zoology

## Abstract

*Tuta absoluta* Meyrick (Lepidoptera: Gelechiidae) is a major pest of tomato worldwide, causing severe leaf and fruit damage and significant yield losses. This study evaluated the effects of calcium and potassium silicates on the biology of *T. absoluta* and tomato productivity under field conditions. Applications of both silicates significantly prolonged immature development to 26.44 and 28.37 days for potassium and calcium silicates, respectively, compared with 22.79 days in the control. The oviposition period decreased to 9.56 and 8.04 days, and fecundity declined to 106.52 and 63.28 eggs, compared with 196.96 eggs in the control. The intrinsic rate of increase (*r*) dropped to 0.126 day^−1^ (potassium) and 0.105 day^−1^ (calcium) versus 0.159 day^−1^ in the control, while the net reproductive rate (*R*_o_) decreased from 118.17 to 63.91 and 35.15, respectively. Both treatments reduced leaf mines, increased fruit weight, and enhanced yield to 18.73 t/ha (potassium) and 23.61 t/ha (calcium), compared with 13.26 t/ha in the control. Postharvest losses were minimized, with calcium silicate showing the lowest weight loss (5.39%) and decay rate (6.77%) after 14 days. These findings demonstrate that calcium and potassium silicates effectively suppress *T. absoluta* populations while improving tomato yield and storability, highlighting their potential for integration into sustainable pest management programs.

**Supplementary Information:**

The online version contains supplementary material available at 10.1038/s41598-026-51521-y.

## **Introduction**

The tomato leafminer, *Tuta absoluta* Meyrick (Lepidoptera: Gelechiidae), is a highly invasive and destructive pest that poses a major threat to tomato production worldwide. Tomato (*Solanum lycopersicum* Mill.), a key vegetable crop cultivated globally for its economic and nutritional value, serves as the principal host of this pest^1,2^. This oligophagous pest attacks the crop at all growth stages by mining leaf tissues and boring into stem and fruits, leading to significant reductions in both yield and quality. Under unmanaged conditions, crop losses caused by *T. absoluta* can reach up to 100%^3,4^.

Its high reproductive potential and strong ability to develop resistance have rendered conventional chemical control increasingly ineffective. Overreliance on synthetic insecticides has further exacerbated resistance development, disrupted populations of beneficial arthropods, and raised public health concerns due to elevated pesticide residues in market-bound produce^5^. These limitations emphasize the urgent need for safer, more sustainable alternatives as part of integrated pest management (IPM) programs^6^.

In this context, plant nutrition and certain exogenous inputs, particularly Silicate -based fertilizers have emerged as promising tools to alter life history traits of herbivorous pests by enhancing host plant resistance^7^. A holistic IPM approach should therefore prioritize methods that strengthen natural plant defenses, conserve beneficial organisms, and maintain ecological balance over exclusive reliance on chemical insecticides^8^.

Among various alternatives, silicate -containing compounds, particularly calcium silicate and potassium silicate have demonstrated potential in reinforcing plant cell walls, increasing phenolic content, and activating endogenous defense pathways^9–11^. These mechanisms can reduce pest fitness and reproductive success, as evidenced in several host–pest systems^12^. Effective pest suppression requires a deep understanding of the pest’s biology and population dynamics. Life tables provide a robust framework for quantifying vital demographic parameters such as survival, development duration, and fecundity across generations of *T. absoluta*, thereby offering valuable insights into pest population trends and treatment efficacy^13^.

However, the specific effects of these silicate-based compounds on *T. absoluta* remain insufficiently studied and warrant further investigation. The effects of calcium silicate and organic mineral fertilizer on *Frankliniella schultzei* Trybom were evaluated, revealing that especially their combined application and repeated use significantly increased nymph mortality and reduced leaf damage on tomato plants^9^. In a study evaluating the effects of magnesium and copper chlorophyllin (Mg-Chl and Cu-Chl) on *T. absoluta*, results showed that these compounds (particularly at higher concentrations) led to 100% reduction in larval tunnels, enhanced tomato plant growth, increased yield, and improved fruit storability^4^. In a study on the effect of silicate against *T. absoluta* under greenhouse conditions, silicate application, significantly reduced the population of immature stages of the pest and effectively enhanced tomato plant resistance^12^. Calcium silicate reduced the oviposition of *Bemisia tabaci* Gennadius (Hemiptera: Aleyrodidae) on bean plants (*Phaseolus vulgaris* L.), in choice and no-choice tests^14^. Potassium silicate (K2SiO3) reduced the yellow mite *Oligonychus sacchari* McGregor population on sugarcane^15^. Application of calcium silicate against *T. absoluta* has been shown to enhance tomato plant resistance and reduce pest-induced damage^16^. Coffee plants (*Coffee* spp.) treated with potassium silicate had a lower infestation by *O. ilicis* McGregor (Tetranychidae) compared to the control due to a reduction in the net reproduction rate values (*R*_*0*_) in life table parameters^17^. The pre-oviposition, oviposition periods, and lifespan of the parent generation of the *Tetranychus urticae* Koch, along with the longevity and oviposition rates of the F1 generation, were adversely impacted by potassium silicate and nanosilica on strawberry plants^18^. Potassium silicate is effective in reducing egg production female *T. urticae* on papaya tree^19^. The use of photosensitizers (Mg-Chl and Cu-Chl) and their nanocomposites with silver and graphene oxide improved growth parameters, increased yield, and enhanced the fruit quality of tomato plants. Additionally, these compounds positively affected the storability of the fruit and showed better effects on plant growth compared to chemical insecticides and the control treatment^4^.

This study aimed to assess the effects of calcium and potassium silicates on the biological performance of *T. absoluta* and to evaluate the field-level impact of these treatments on tomato plant growth, yield, and postharvest quality under field conditions.

## Materials and methods

### Preparation of silicate treatments

Three treatment solutions were prepared: potassium silicate (K_2_SiO_3_) and calcium silicate (CaSiO_3_), each at a concentration of 30 mL per L of water, and deionized water as a silica-free control.

### Experimental design

The experiment was conducted at the Urmia University Faculty of Agriculture experimental station (Urmia, Iran). The tomato cultivar ‘Robin’, commonly grown in Iran, was selected for field evaluation in a village near Nazlu. Tomato plants were transplanted with 30 cm between plants within rows and 60 cm between rows (80 cm long and 60 cm wide/row). Each experimental plot consisted of 4 rows × 10 plants (plot size: 1.8 m × 3 m = 5.4 m^2^). The experiments were arranged in a randomized complete block design (RCBD) with four replicates per treatment. All plots received standard agricultural practices including irrigation and fertilization. The first application of treatments was performed at the onset of flowering and was followed by two additional applications at )10-day intervals ((total of three applications). For each treatment, the silicate solution (30 mL per L water) was uniformly sprayed over the entire plot using an electric knapsack sprayer (calibrated at 400 L ha^−1^), ensuring thorough coverage of all aerial parts. Spraying was carried out in the evening under calm weather conditions to minimize drift and enhance absorption. Plant samples were investigated before and after the treatment on the 30, 35, and 40 days.

### Insect release within field cages

To prevent the entry or escape of pests and natural enemies, each experimental plot was covered, after transplanting tomato seedlings, with a mesh cage measuring approximately 1.20 × 1.20 × 1.20 m (length × width × height). Inside each cage, a predetermined 50 of adult *T. absoluta*, reared in the laboratory and ready for oviposition, were uniformly released onto the plants. This procedure was carried out prior to the first spraying to ensure a uniform infestation level across all plots.

### Data recorded

#### Reduction percentage in tunnels of ***T. absoluta***

In each plot (potassium silicate, calcium silicate and control), 40 plants were randomly selected in each treatment (10 plants per blocks) and examined for the presence of infested plants (leaves, stem and fruit). The number of larval tunnels per plant was recorded, and the efficacy of each treatment was expressed as a percentage reduction in infestation. This was calculated based on the mean tunnel counts in treated versus untreated control plants, using the Henderson and Tilton^20^. formula, which accounts for changes in both.

$$\:\mathrm{E}\mathrm{f}\mathrm{f}\mathrm{i}\mathrm{c}\mathrm{a}\mathrm{c}\mathrm{y}\:\mathrm{o}\mathrm{f}\:\mathrm{t}\mathrm{r}\mathrm{e}\mathrm{a}\mathrm{t}\mathrm{m}\mathrm{e}\mathrm{n}\mathrm{t}\mathrm{\%}=1-\left(\frac{\mathrm{T}\mathrm{a}\mathrm{*}\mathrm{C}\mathrm{b}}{\mathrm{C}\mathrm{a}\mathrm{*}\mathrm{T}\mathrm{b}}\right)*100$$

where Ta is the all larval tunnels per 40 plants in treatment after application. Tb is the all larval tunnels per 40 plants in treatment before application. Ca is the all larval tunnels per 40 plants in Control after application. Cb is the all larval tunnels per 40 plants in Control before application.

### Fruit yield parameters

At 60 days after transplanting, fruit yield parameters were evaluated. In each experimental plot, 20 tomato plants were randomly selected. All marketable fruits (approximately 80% ripeness) were harvested from each plant. The total number of fruits per plant and their total fresh weight were recorded using a digital balance with 0.01 g precision. The average fruit weight was calculated by dividing the total fruit weight by the fruit count.

The estimated yield per hectare (tons ha⁻¹) was calculated by multiplying the average fruit yield per plant by the planting density, and converting the result from kilograms to tons. This calculation approach follows standard agronomic practices for tomato production yield estimation.

### Yield and postharvest storability

At commercial maturity, 20 uniform and healthy tomato fruits (approximately 80% red coloration) were randomly selected from each experimental plot. These fruits were stored in plastic boxes at 10 °C and 70% relative humidity for 14 days. Fruit quality was assessed at two time points: 7 and 14 days after storage. The weight of the fruits was measured before and after storage to calculate the percentage of weight loss using the formula.

Weight loss (%): measured by initial and subsequent weight of fruits using

$$\:\mathrm{W}\mathrm{e}\mathrm{i}\mathrm{g}\mathrm{h}\mathrm{t}\:\mathrm{l}\mathrm{o}\mathrm{s}\mathrm{s}\:\mathrm{\%}=\left(\frac{\mathrm{W}\mathrm{e}\mathrm{i}\mathrm{g}\mathrm{h}\mathrm{t}\:\mathrm{a}\mathrm{t}\:\mathrm{D}\mathrm{a}\mathrm{y}\:0-\mathrm{W}\mathrm{e}\mathrm{i}\mathrm{g}\mathrm{h}\mathrm{t}\:\mathrm{a}\mathrm{t}\:\mathrm{D}\mathrm{a}\mathrm{y}\:\mathrm{X}}{\mathrm{W}\mathrm{e}\mathrm{i}\mathrm{g}\mathrm{h}\mathrm{t}\:\mathrm{a}\mathrm{t}\:\mathrm{D}\mathrm{a}\mathrm{y}\:0}\right)*100$$

Fruits exhibiting signs of shriveling, rotting, or physiological damage were separated, weighed, and their proportion relative to the total fruit weight was recorded as the percentage of decay.

Decay rate (%): weight of fruits showing visual decay (shrivel, rot) divided by total fruit weight

$$\:\mathrm{D}\mathrm{e}\mathrm{c}\mathrm{a}\mathrm{y}\:\mathrm{r}\mathrm{a}\mathrm{t}\mathrm{e}\:\mathrm{\%}=\left(\frac{\mathrm{W}\mathrm{e}\mathrm{i}\mathrm{g}\mathrm{h}\mathrm{t}\:\mathrm{o}\mathrm{f}\:\mathrm{d}\mathrm{e}\mathrm{c}\mathrm{a}\mathrm{y}\mathrm{e}\mathrm{d}\:\mathrm{f}\mathrm{r}\mathrm{u}\mathrm{i}\mathrm{t}}{\mathrm{T}\mathrm{o}\mathrm{t}\mathrm{a}\mathrm{l}\:\mathrm{f}\mathrm{r}\mathrm{u}\mathrm{i}\mathrm{t}\:\mathrm{w}\mathrm{e}\mathrm{i}\mathrm{g}\mathrm{h}\mathrm{t}}\right) \times100$$.

### The effects of calcium silicate and potassium silicate treatments on the SPAD chlorophyll index

The effects of calcium silicate and potassium silicate treatments on the SPAD (Soil Plant Analysis Development) chlorophyll index of tomato leaves were investigated. Chlorophyll content of plants treated with calcium silicate, potassium silicate, and water (control) was measured using a chlorophyll meter (SPAD, Minolta 502, Japan) at 5, 10, and 15 days after spraying. The measurement principle was based on the transmission of light wavelengths in the red range (650 nanometers) and infrared range (950 nanometers) through the leaf. Chlorophyll absorbs red light but transmits infrared light. Based on the difference in light transmission between these two wavelengths, the device calculates a value (SPAD) that correlates closely with the total chlorophyll content.

### Life table parameters of *T. absoluta*

Specimens of *T. absoluta* were collected from tomato fields and reared in the laboratory on healthy tomato plants under controlled conditions. Adult moths mated and oviposited on the plants. Eggs were monitored daily to assess larval development and population establishment. The colony was maintained for several generations to ensure a stable population for experiments. Tomato plants (*Solanum lycopersicum* L.) were grown in 20-L plastic pots under controlled laboratory conditions and treated with potassium silicate, calcium silicate, and water as control. The first application was performed at the onset of flowering in tomato plants, followed by reapplications every 10 days. For each treatment, 5 mL of solution was uniformly sprayed on each plant to ensure complete leaf coverage and effective absorption. The silicate solution was prepared by mixing 30 mL of concentrate in L of water. These treated plants served as oviposition substrates for evaluating the life table parameters of *Tuta absoluta.*

For each treatment, 40 mated and gravid females of *T. absoluta* were released onto the treated tomato plants to allow oviposition. Leaves containing eggs were enclosed individually in plastic containers (12 × 8 × 8 cm), with lids covered by organza mesh to allow ventilation while preventing mite escape. Eggs were monitored daily. Due to the endophytic development of larval stages within the leaf tissue, the duration of the entire immature period (egg, overall larval stage, and pupal stages) until adult emergence was recorded. Newly emerged adults from each treatment were sexed, and one male and one female from the same treatment group were paired and placed individually in oviposition cages supplied with leaflets from similarly treated tomato plants. These pairs were monitored daily for longevity, fecundity, and reproductive parameters until the death of the female. Life history parameters recorded included pre-oviposition period, oviposition period, total fecundity, egg viability, adult longevity, and population growth parameters such as intrinsic rate of increase (*r*), net reproductive rate (*R*_o_), finite rate of increase (*λ*), and mean generation time (*T*). All experiments were conducted under controlled conditions: 25 ± 2 °C, 65 ± 5% RH, and a 16:8 h light: dark photoperiod.

### Statistical analysis

Data were statistically analyzed using generalized linear models (GLM) univariate method. Mean comparisons for field and storage-related traits, including, damage reduction, weight loss, decay rate and chlorophyll index, were performed using Tukey’s honest significant difference (HSD) test at *P* < 0.05 via SPSS software (version 27)^21^. The TWOSEX-MSChart software^22^, as a tool for calculating the demographic parameters of insects, was used to analyze the raw data regarding development and reproduction, as well as to calculate population parameters for all individuals using the age-stage, two-sex life table method^23,24^. For life table parameters, differences among treatments were compared using the bootstrap procedure implemented in TWOSEX-MSChart with 100,000 resampling iterations; these comparisons were conducted as pairwise contrasts among independent treatment groups. Analysis of the population growth trend was performed using TIMING-MSChart statistical software^25^. The figures were depicted by SigmaPlot 15^26^.

## Results

### Effect of calcium and potassium silicates on the population of *Tuta absoluta*.

Data analysis revealed that block effects were not significant (*P* > 0.05), whereas significant differences among treatments were observed. At 30, 35, and 40 days after the third foliar application, tomato plants treated with calcium and potassium silicates showed a reduction in the number of leaf mines caused by *T. absoluta*. A reduction in mining activity was observed at 30 days after treatment and persisted through 35 and 40 days. No statistically significant differences were detected between the two silicate treatments at any time point. The percentage reduction increased progressively with time, reaching up to 97% in the calcium silicate treatment. In contrast, water-treated control plants showed a continuous increase in mine formation during the same period (Table 1).

**Table 1:** Impact of calcium and potassium silicates on reducing damage caused by *Tuta absoluta* under field condition.

Treatments	Before spray	Mean number of newly-tunnels and reduction percentages					
		Day post first spray					
		30		35		40	
		Mean ± SE	Reduction (%)	Mean ± SE	Reduction (%)	Mean ± SE	Reduction (%)
Calcium silicates	2.12 ± 0.11a	1.07 ± 0.10^b^	36.58	0.40 ± 0.07^b^	83.07	0.10 ± 0.04^b^	97.33
Potassium silicates	1.90 ± 0.10a	1.15 ± 0.10^b^	24.12	0.75 ± 0.09^b^	64.51	0.27 ± 0.07^b^	91.79
Control	2.225 ± 0.14a	1.77 ± 0.14^a^	–	2.47 ± 0.17^a^	−	3.92 ± 0.23^a^	−
F value	2.069	9.75		83.78		203.37	
P value	Ns*	0.001		0.001		0.001	

Means within a column followed by the same letter are not significantly different using tukey’s Multiple Range Test. Small letters indicate to significant differences between treatments.

*= non-significant.

*N* = 40.

In insignificant.

### Effect of calcium and potassium silicate treatments on tomato fruit yield

Tomato plants treated with calcium and potassium silicates consistently produced heavier fruits and higher total yields compared to the control group. Data analysis revealed that block effects were not significant (*P* > 0.05), but the significant differences on treatments were observed. The increase in fruit production was statistically significant (*P* < 0.05), indicating the effectiveness of calcium and potassium silicate in enhancing tomato productivity under field conditions.

The cumulative fruit yield per plant in the calcium silicate treatment reached an average of 11.33 kg, followed by 8.99 kg in the potassium silicate treatment, both exceeding the general range reported under typical conditions. In contrast, control plants yielded an average of 6.36 kg per plant during two mounts. Based on a planting density of about 2083 plants ha^−1^, the estimated yield per hectare was calculated. The highest yield was observed in calcium silicate-treated plots (23.61 t ha^−1^), followed by potassium silicate (18.73 t ha^−1^), both significantly higher than the control (13.26 t ha^−1^) (Table 2).

**Table 2:** Effects of calcium and potassium silicate treatments on tomato fruit yield parameter.

Treatment	Avg. fruit weight (g)	Yield per plant (kg)	Estimated yield (t/ha)
Calcium silicate	287.19 ± 3.44^a^	11.33 ± 0.15^a^	23.61
Potassium silicate	256.49 ± 2.52^b^	8.99 ± 0.14^b^	18.73
Control	177.03 ± 2.71^c^	6.36 ± 0.13^c^	13.26
F value	313.41	287.62	
P value	0.001	0.001	

Means within columns followed by the same letter are not significantly different at *P* < 0.05 using Tukey’s multiple range test. lowercase letters indicate differences among treatments.

### Weight loss and decay during storage

**Fig. 1:**
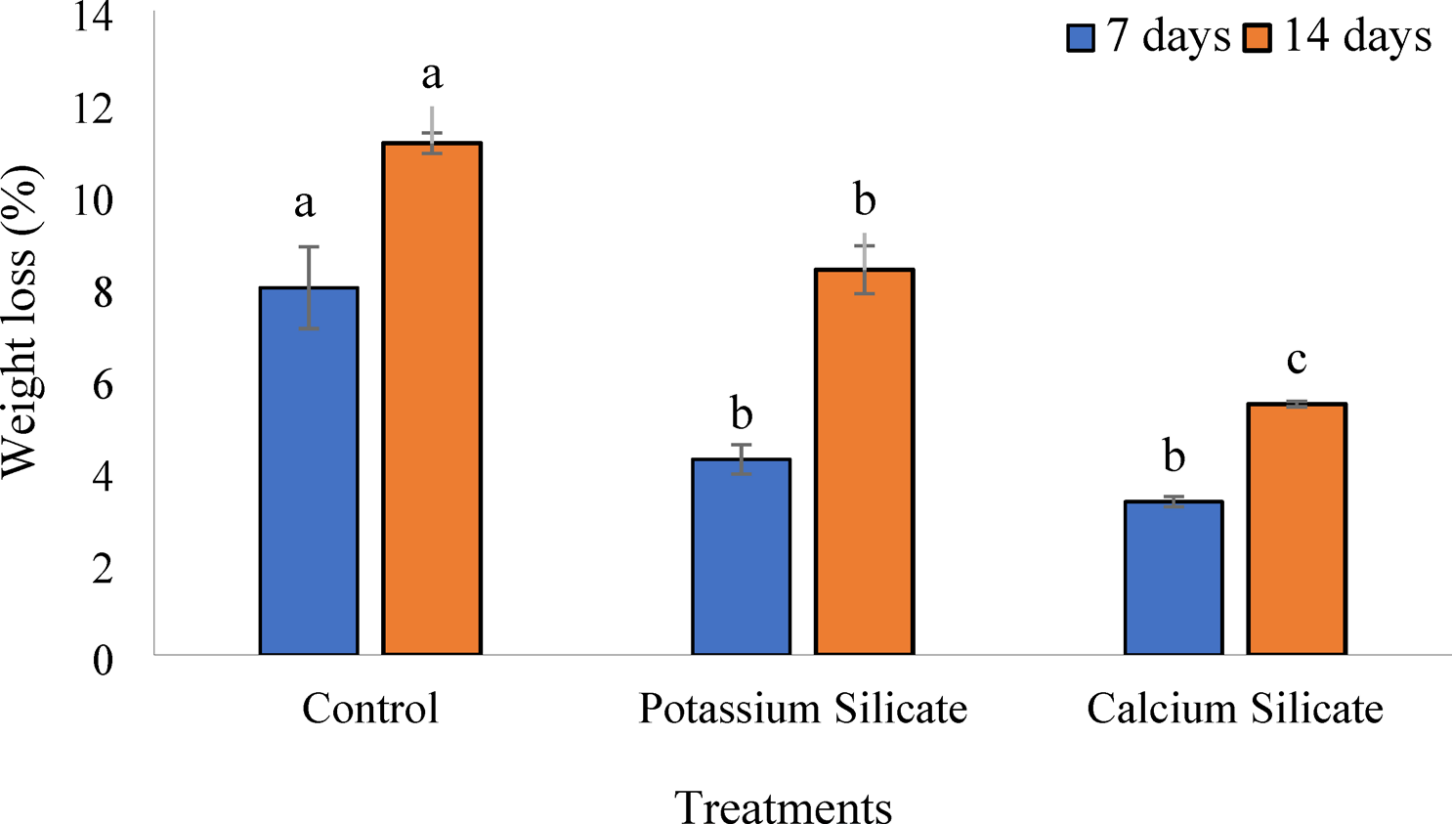
Effect of potassium silicate, calcium silicate on weight loss % of tomato.

**Fig. 2:**
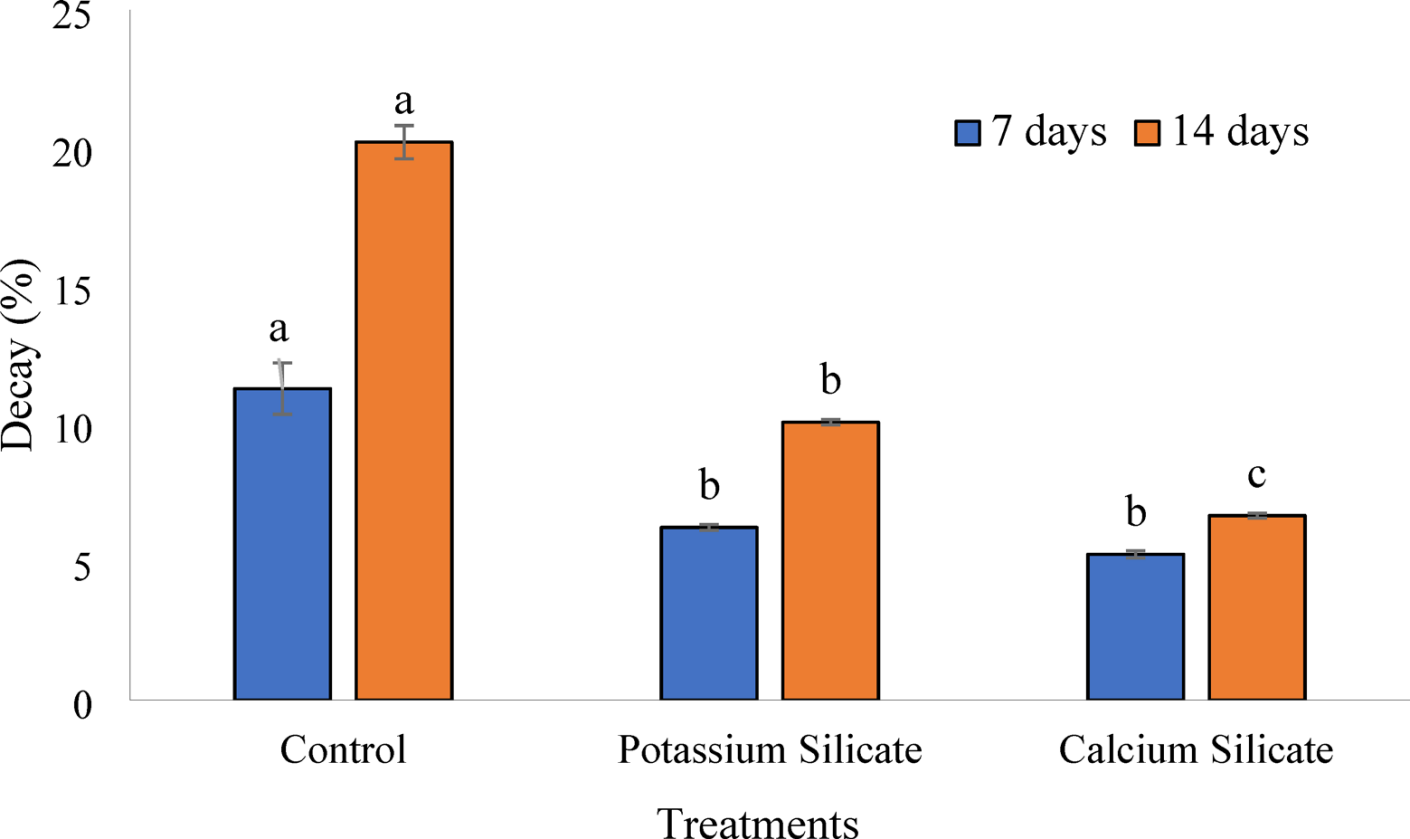
Effect of potassium silicate, calcium silicate on decay % of tomato.

In experiment on weight loss and decay during storage analysis revealed that block effects were not significant (*P* > 0.05), but the significant differences on treatments were observed. After 7 and 14 days of storage, both potassium and calcium silicate treatments significantly reduced (F _(2, 6)_ = 131.73, *P* = 0.001; F _(2, 6)_ = 28.06, *P* = 0.001) the percentage of fruit weight loss compared to the control (Fig. 1). The lowest weight loss was recorded in calcium silicate treatment (5.45% at day 14), followed by potassium silicate (8.37% at day 14), while the control showed the highest weight loss (11.12%). Similarly, the decay percentage was significantly reduced in treated fruits after 7 and 14 days (F _(2, 6)_ = 556.72, *P* = 0.001; F _(2, 6)_ = 54.36, *P* = 0.001) compared to the control. At 14 days, the calcium silicate treatment had the lowest decay rate (6.68%) compared to potassium silicate (10.06%) and the untreated control (20.21%). The improvement in storability may be attributed to the enhanced physiological resistance conferred by silicate fertilizer application (Fig. 2).

### Chlorophyll index (SPAD)

**Fig. 3:**
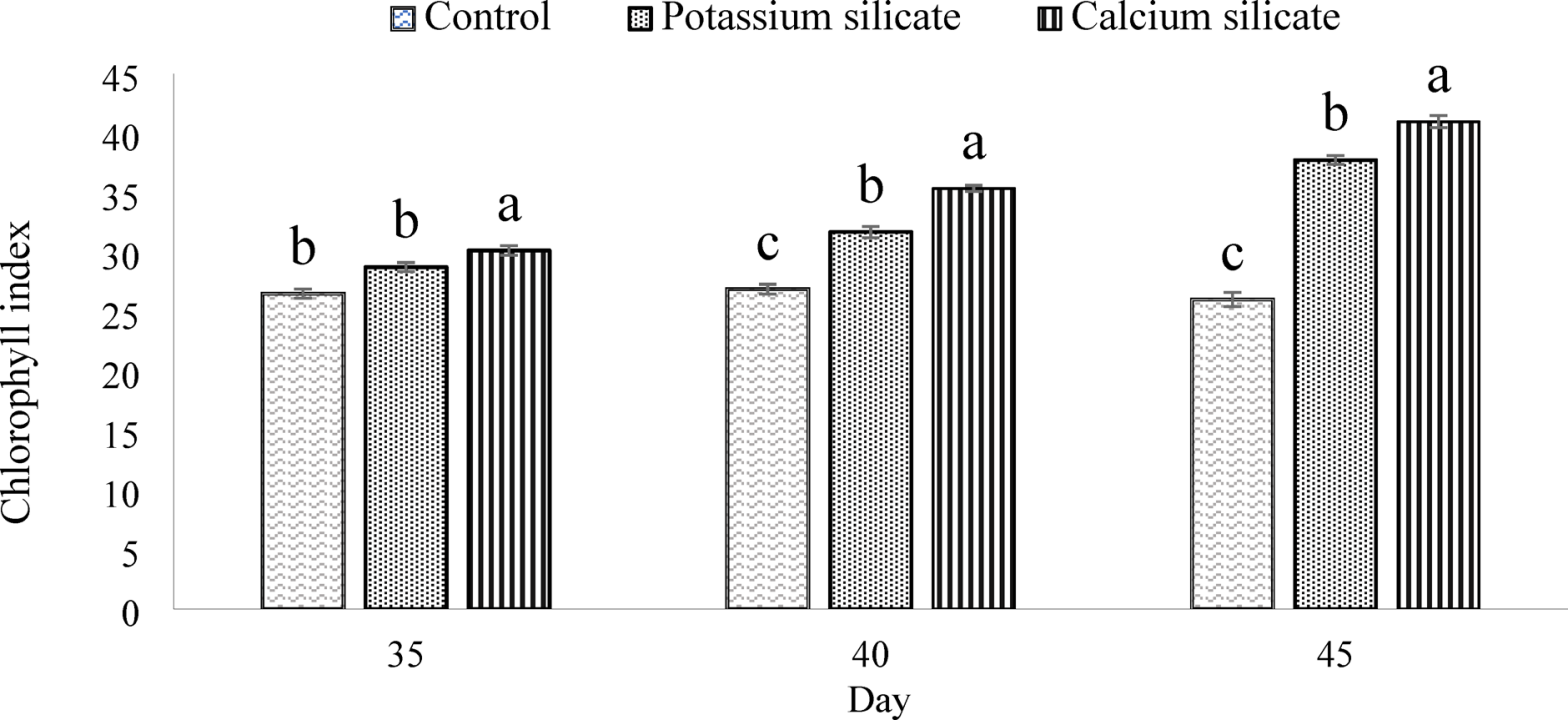
Mean of leaf chlorophyll index treated by potassium silicate, calcium silicate, and control treatments at 95% confidence level with Tukey test. The columns with the same letters indicate non-significant differences between treatments.

Data analysis indicated that block effects were not significant (*P* > 0.05); however, significant treatment effects were detected. Chlorophyll content differed significantly among treatments at 30, 35, and 40 days after spraying (F _(2, 6)_ = 31.07, *P* = 0.001; F _(2, 6)_ = 70.45, *P* = 0.001; and F _(2, 6)_ = 174.84, *P* = 0.001, respectively) (Fig. 3). Comparison of treatment means revealed that the SPAD chlorophyll index was highest in the calcium silicate treatment, with significant differences at the 95% confidence level compared with potassium silicate and control treatments (Fig. 3).

### Life table parameters of *T. absoluta*

The biological parameters of *T. absoluta* population on tomato plants treated with calcium silicate and potassium silicate compared with control plants are presented in Table 3. The biological parameters of the *T. absoluta* were significantly affected by the treatments. The minimum life span of the adult female and the fecundity of the *T. absoluta* were observed on the calcium silicate treatment. The oviposition period of female pests in the control treatment was longer than those in the calcium silicate and potassium silicate treatments.

**Table 3:** Duration of *Tuta absoluta* life stages and lifetime fecundity on plants treated with calcium and potassium silicate.

Different life stages (day) and fecundity	Treatment		
	Control	Potassium silicate	Calcium silicate
Egg (♀+♂)	4.44 ± 0.08^a^	4.52 ± 0.09^a^	4.55 ± 0.09^a^
Larve (♀+♂)	11.14 ± 0.11^c^	14.00 ± 0.14^b^	15.05 ± 0.23^a^
Pupa (♀+♂)	7.19 ± 0.11^c^	7.87 ± 0.16^ab^	8.54 ± 0.15^a^
Developmental time (day)	22.79 ± 0.14^c^	26.44 ± 0.23^b^	28.37 ± 0.27^a^
Adult female longevity (day)	15.15 ± 0.21^a^	12.37 ± 0.21^b^	10.26 ± 0.12^c^
Adult male longevity (day)	15.00 ± 0.26^a^	11.92 ± 0.26^b^	10.10 ± 0.18^b^
APOP^*^	1.26 ± 0.10^a^	1.56 ± 0.10^b^	1.28 ± 0.11^a^
TPOP^**^	24.11 ± 0.21^b^	28.07 ± 0.25^a^	29.84 ± 0.37^a^
Oviposition days	12.93 ± 0.15^a^	9.56 ± 0.25^b^	8.04 ± 0.20^c^
Fecundity (no. eggs)	196.96 ± 2.83^a^	106.52 ± 2.99^b^	63.28 ± 1.53^c^

*APOP: Adult Pre-Ovipositional Period (from Incubation to first oviposition).

**TPOP: Total Pre-Ovipositional Period (from egg to first oviposition).

### Population growth parameters of *T. absoluta*

The population growth parameters of *T. absoluta* on tomato plants treated with calcium silicate, potassium silicate, and control are shown in Table 4. The growth parameters of the *T. absoluta* population, including Gross Reproductive Rate (*GRR*), net reproductive rate (*R*_0_), intrinsic rate of increase (*r*) and the finite rate of increase (λ) were significantly affected in the calcium silicate and potassium silicate compared to the control (Table 4).

**Table 4:** Mean (± SE) life table parameters for *Tuta absoluta* on plants treated to calcium and potassium silicate application. Means were separated with paired bootstrap test (*P* < 0.05) and standard errors by bootstrap with 100,000 samples. Values bearing different letters were significantly different among treatments.

Parameters	Treatment		
	Control	Potassium silicate	Calcium silicate
*GRR* (offspring)	144.95 ± 18.17^a^	82.92 ± 9.03^b^	57.81 ± 7.74^c^
*R*_0_ (offspring)	118.17 ± 14.46^a^	63.91 ± 7.98^b^	35.15 ± 4.75^c^
*r* (day^− 1^)	0.159 ± 0.004^a^	0.126 ± 0.004^b^	0.105 ± 0.004^c^
*λ* (day^− 1^)	1.172 ± 0.005^a^	1.135 ± 0.004^ab^	1.110 ± 0.004^b^
*T* (day)	29.93 ± 0. 21^a^	37.78 ± 0.26^b^	33.88 ± 0.33^c^

### Age-specific survival, fecundity, and reproductive performance of *Tuta absoluta* under calcium and potassium silicate applications

**Fig. 4:**
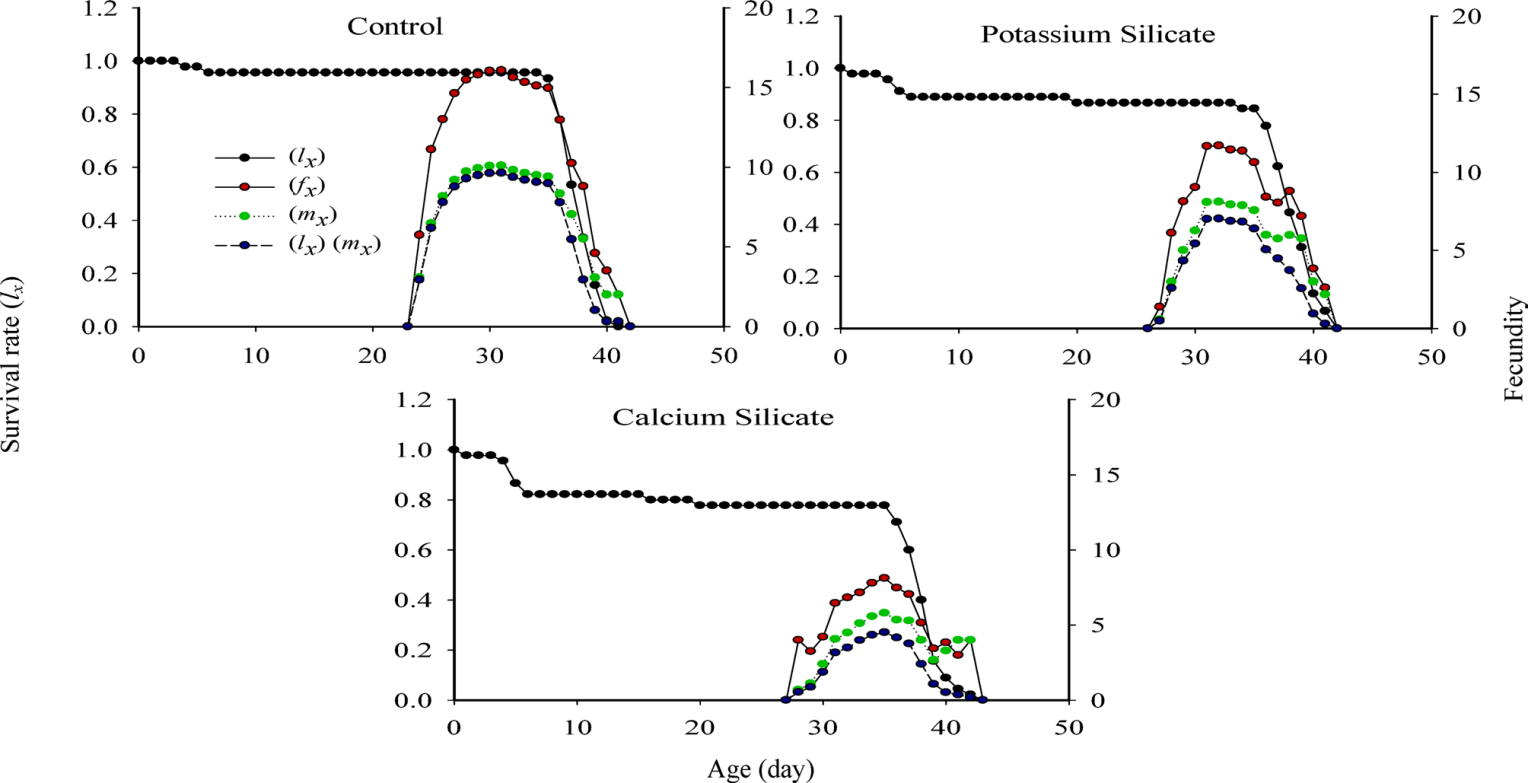
Age-specific survival rate (*l*_*x*_), age-specific fecundity (*m*_*x*_), and age-stage-specific fecundity (*f*_*x*_) of *Tuta absoluta* following calcium silicate and potassium silicate application to enhance plant resistance.

The age-specific survival rate (*l*_*x*_), age-specific fecundity (*m*_*x*_), and age-stage-specific fecundity (*f*_*x*_) of *T. absoluta* on tomato plants treated with calcium and potassium silicate decreased compared to the control (Fig. 4). The age-specific fecundity (*m*_*x*_), which represents the number of female offspring produced per female at a given age (*x*), was lower under both treatments. The highest *m*_*x*_ value was observed in the control group, reaching 10.09 eggs per female on day 30, whereas it decreased to approximately 8.07 and 4.05 eggs per female under potassium silicate and calcium silicate treatments, respectively.

**Fig. 5:**
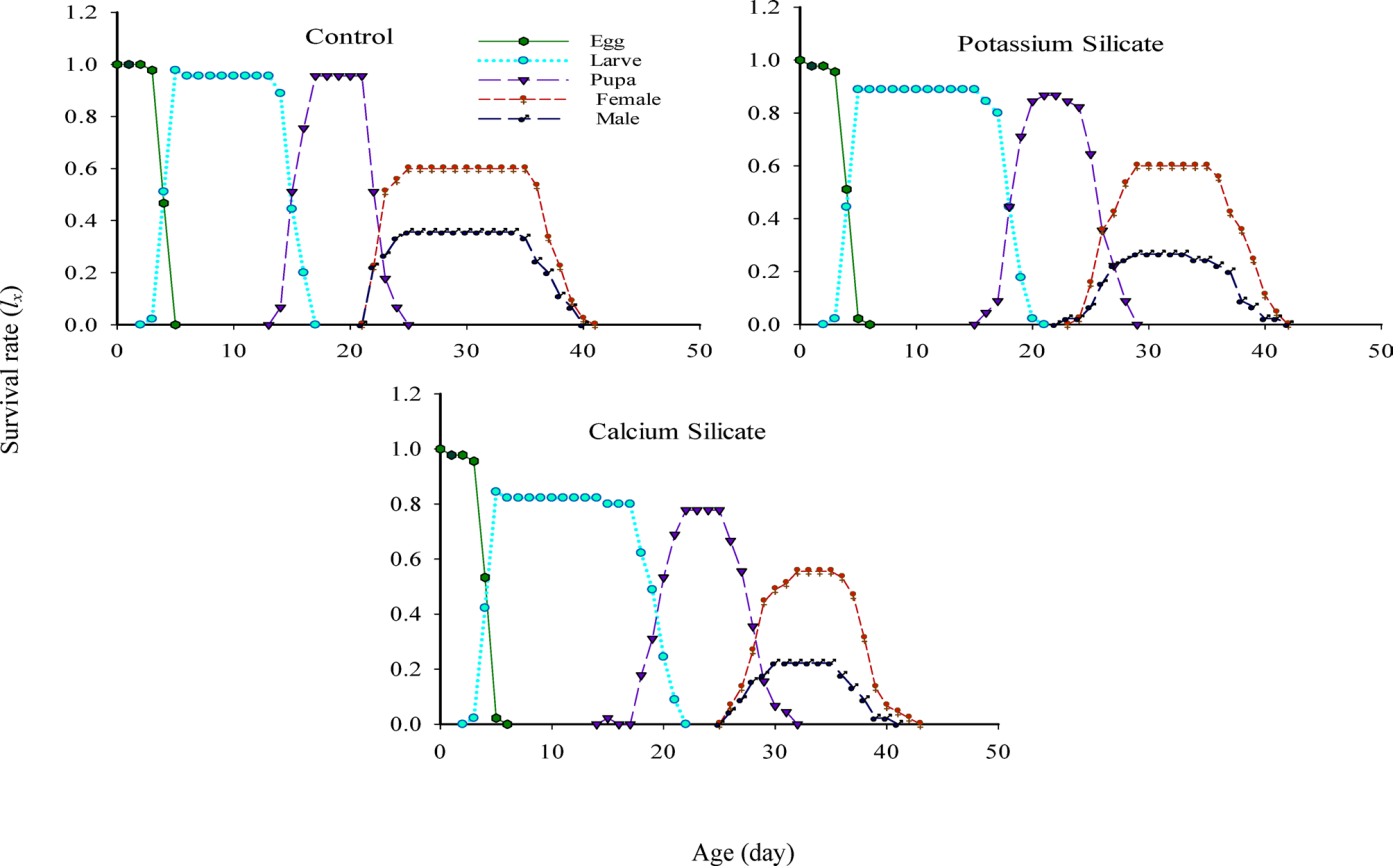
Age-stage-specific survival rate (*S*_*xj*_) of *Tuta absoluta* following calcium silicate and potassium silicate application to enhance plant resistance.

The stage differentiation and survivorship curves of *T. absoluta* on tomato plants treated with calcium and potassium silicate application are shown in Fig. 5. The female survival rate (*l*_*x*_) is lower in calcium and potassium silicate treatments compared with control.

The age-specific life expectancy (*e*_xj_) of *T. absoluta* adults decreased under both treatments compared to the control. This reduction was more pronounced in the calcium silicate treatment than in the potassium silicate treatment, indicating a stronger effect of calcium on reducing adult longevity. The (*e*_xj_) during larval stages increased under both treatments, indicating an improvement in larval survival compared to the control.

**Fig. 6:**
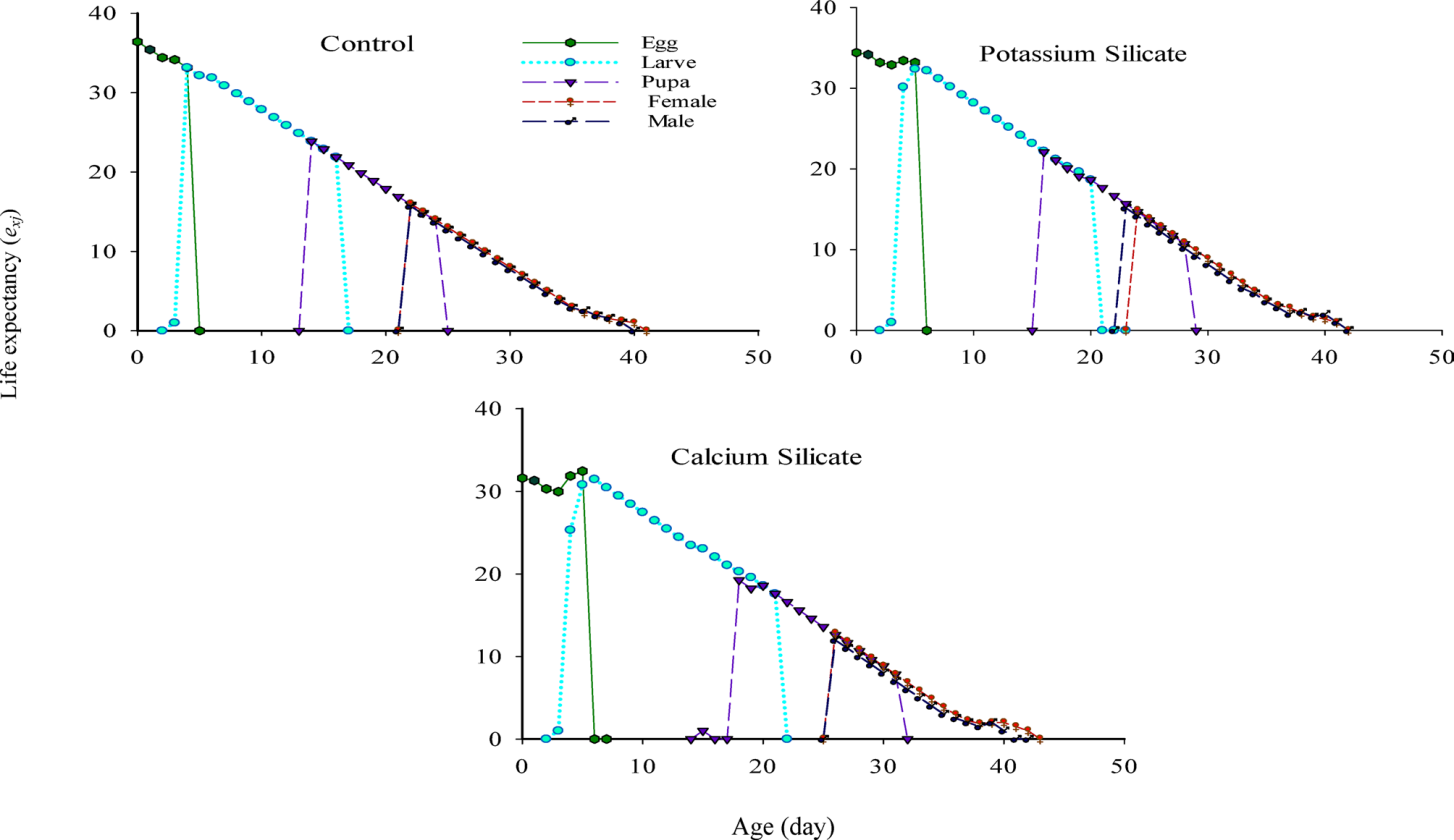
Age-stage-specific life expectancy (*e*_*xj*_) of *Tuta absoluta* following calcium silicate and potassium silicate application to enhance plant resistance.

The life expectancy (*e*_*xj*_) of each age-stage of *T. absoluta* female in the treatments of potassium silicate, calcium silicate, and control were 14.88, 12.84 and 16.00 days, respectively, as plotted in Fig. 6. The *e*_*xj*_ of calcium silicate was the lowest for all stages.

**Fig. 7:**
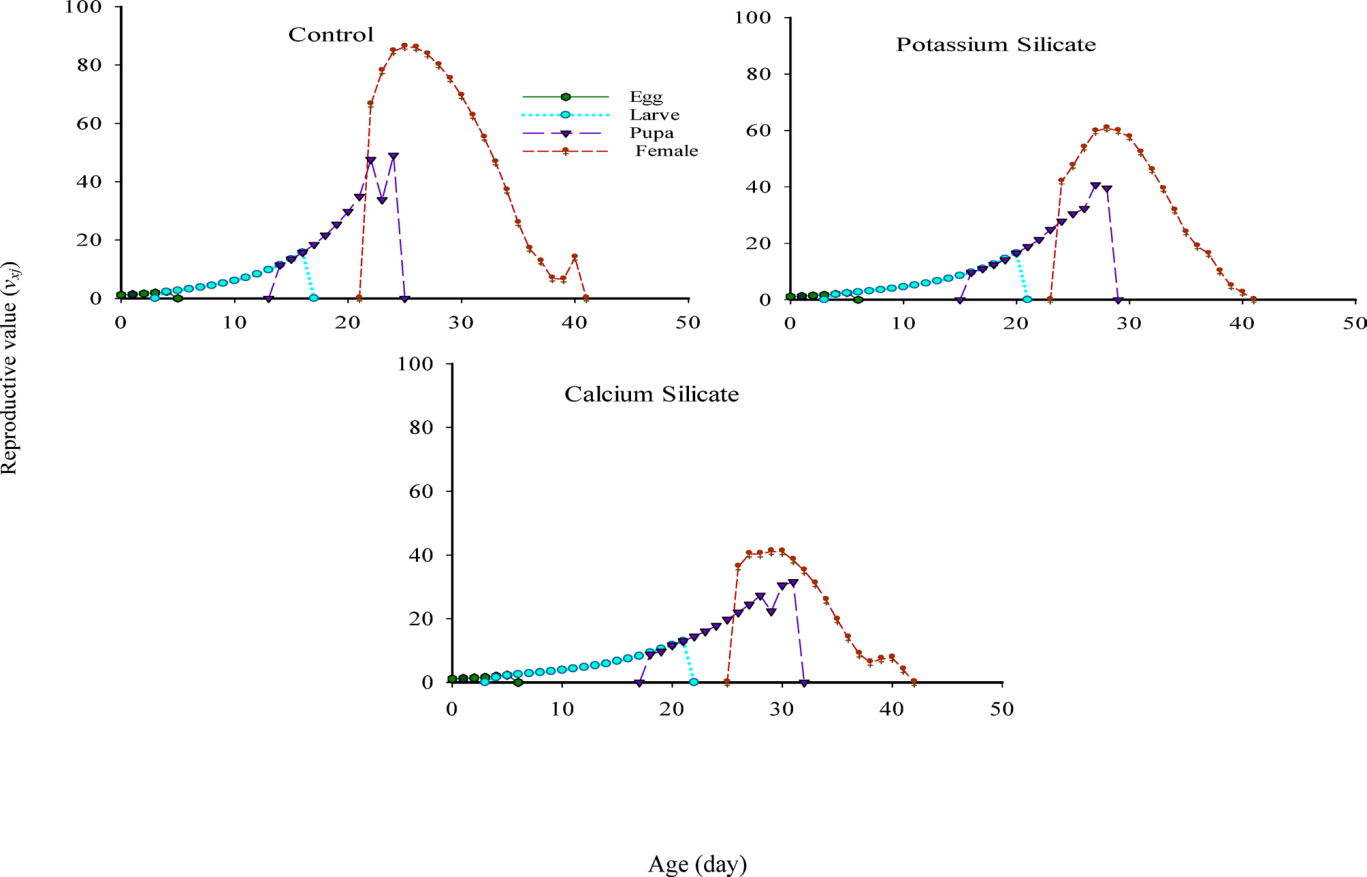
Age-stage-specific reproductive value (*v*_*xj*_) of *Tuta absoluta* on plants treated with calcium and potassium silicate application.

The age–stage-specific reproductive value (*v*_*xj*_) represents the contribution of individuals at a specific age and developmental stage to the future population (Fig. 7). According to the results, the reproductive value of both adult females and immature stages decreased under the treatments compared to the control. The highest reproductive value was observed in the control group (86.32 offspring per female), while the potassium silicate treatment was (60.85 offspring per female) and the lowest value was recorded in the calcium silicate treatment (9.33 offspring per female).

### The population growth trends of total population size of *T. absoluta*

**Fig. 8:**
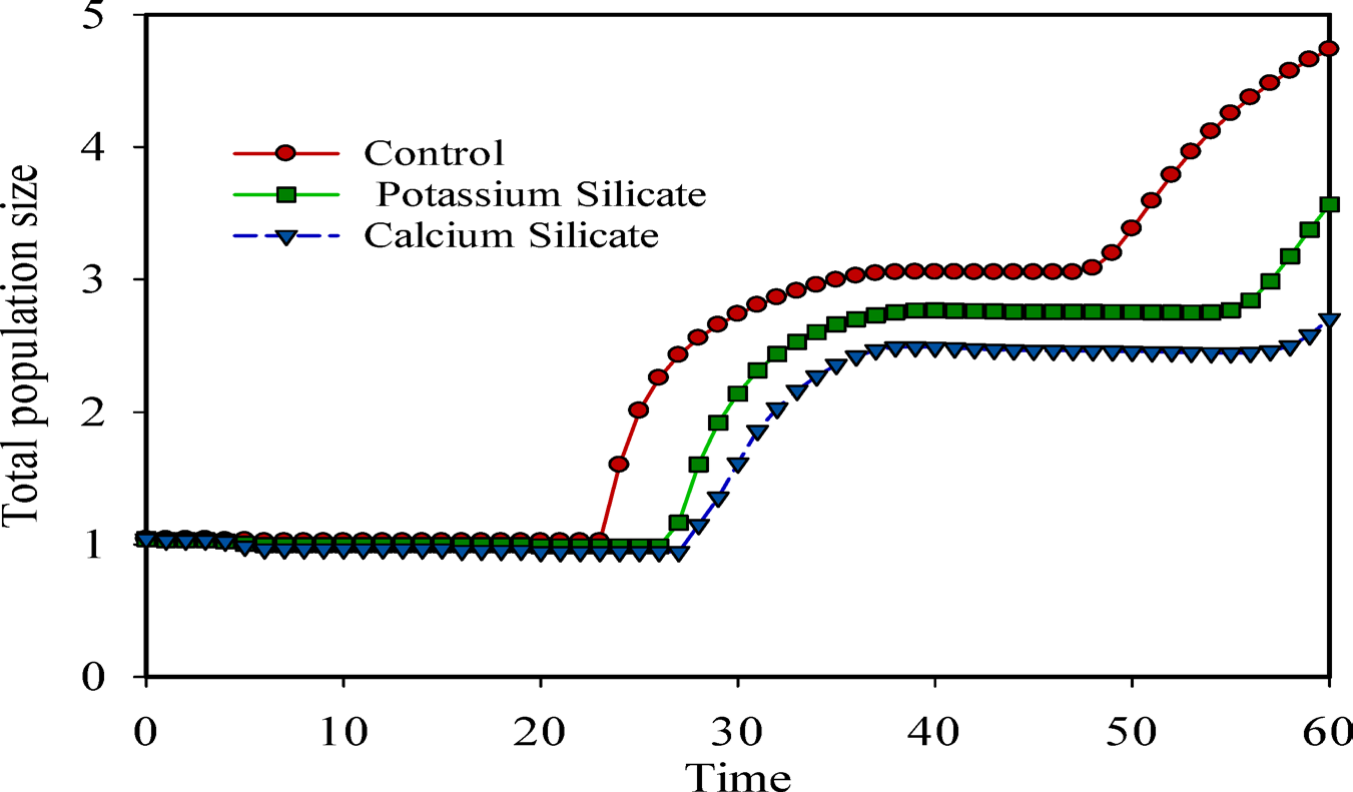
The growth trends of the total population of the leaf miner, *Tuta absoluta*, on plants treated to potassium silicate, calcium silicate, and control.

The effects of calcium silicate and potassium silicate treatments on the population growth trends of different developmental stages of the tomato leaf miner are presented in Fig. 8. Population growth was projected for each treatment based on an initial cohort of 45 eggs over a 60-day period. As illustrated, population growth proceeded at a relatively similar rate during the early stages, particularly the egg stage. However, in the later developmental stages, the growth rate slowed noticeably under the influence of the potassium silicate and calcium silicate.

## Discussion

The beneficial effects of silicate compounds, including calcium and potassium silicates, on crop performance and quality have been well documented^11,27^. In this study, tomato plants treated with calcium and potassium silicates showed a significant reduction in feeding tunnels per leaf, with decreases of 97.33% and 91.79%, respectively, at 40 days post-treatment compared to the control. These treatments also resulted in heavier fruits and higher yields per plant, with extrapolated yields of 23.61 t/ha for calcium silicate and 18.73 t/ha for potassium silicate, compared to 13.26 t/ha in untreated plants. Postharvest evaluations revealed that calcium and potassium silicate treatments effectively reduced fruit deterioration during storage. Notably, calcium silicate treatment produced the lowest weight loss (5.39%) and decay rate (6.77%) after 14 days, emphasizing its potential to improve fruit storability. Furthermore, SPAD chlorophyll index measurements indicated enhanced chlorophyll content in treated plants compared to the control.

These findings align with previous research demonstrating that different silicate sources (including calcium, potassium, and sodium silicates) increased silicate content in tomato leaves, improved marketable yield, and reduced fruit cracking^22^. Similarly, foliar application of potassium silicate under deficit irrigation (50% ET_o_) significantly increased stem height, leaf chlorophyll content, fruit yield, and water use efficiency in tomato plants^29^. Silicate foliar sprays have also been reported to improve the nutritional profile of tomato fruits^27^. This study also demonstrated that calcium and potassium silicate treatments prolonged the larval period, decreased fecundity, and suppressed the population growth of *T. absoluta*. The intrinsic rate of increase (*r*) declined to 0.126 day^−1^ and 0.105 day^−1^ for potassium and calcium silicate treatments, respectively, compared to 0.159 day^−1^ in the control. Similarly, the net reproductive rate (*R*_o_) decreased from 118.17 in the control to 63.91 and 35.15 with potassium and calcium silicate treatments, respectively. These treatments further reduced life expectancy and reproductive value, indicating a strong negative impact on pest population growth. These effects are likely due to the combined physiological and mechanical actions of silicate, including anti-feeding activity that reduces oviposition and disrupts larval establishment, highlighting the potential of calcium and potassium silicates as valuable components in integrated pest management for tomatoes.

silicate -based compounds have been proposed as effective agents for managing *T. absoluta* due to their larvicidal and feeding-deterrent properties, as well as their ability to enhance plant resistance mechanisms^30,31^. However, direct evidence for silicate accumulation as silica deposits in leaf tissues as a resistance mechanism remains limited^32,33^. Silicate application is known to strengthen leaf tissues and interfere with insect feeding and development^34^, resulting in reduced oviposition, lower larval survival and weight, shorter larval and pupal durations, and overall suppressed pest fitness^35,36^.

In a field study, nanosilica (NS) and jasmonic acid (JA) were evaluated for *T. absoluta* control under commercial tomato cultivation. Nanosilica and jasmonic acid showed the greatest reduction in larval leaf mining rates, indicating strong insecticidal and anti-feeding activity^37^. Similarly, foliar application of calcium silicate, alone or combined with benzothiadiazole-S-methyl (BTH), increased nymphal mortality and prolonged development from nymph to adult in *B. tabaci* on cucumber, effects attributed to induced plant defense responses^32^. Silicon treatments stimulate defense-related enzymes such as chitinase, peroxidase, 1,3-glucanase, polyphenol oxidase, phenylalanine ammonia-lyase, protease, and lipoxygenase in crops including cotton, tomato, and wheat^32^. These enzymes facilitate the metabolism of phenolic compounds like lignin, which increases cell wall rigidity and reduces the nutritional quality and digestibility of plant tissues, ultimately limiting pest population growth^33,38^. Additionally, calcium silicate application, alone or combined with organic-mineral fertilizers, significantly reduced the population density of *F. schultzei* and feeding damage on tomato foliage^9^. Population growth modeling in this study further confirmed that calcium silicate effectively suppressed *T. absoluta* growth over a 60-day period, with treated plants showing a significant reduction in intrinsic rate of increase compared to controls. Similar results have been observed in cucumber plants where calcium silicate, with or without BTH, increased mortality and extended development in *B. tabaci* by enhancing plant defense substances. Silicon-induced enzyme activation plays a critical role in pest resistance by altering plant biochemistry to deter insect development^32,33,38^.

Overall, while the results of this study support the role of silicate-based treatments in reducing survival, prolonging development, and decreasing the reproductive capacity of *T. absoluta*, the underlying mechanisms should be interpreted cautiously. The observed effects are most consistent with antibiosis, whereas the contribution of antixenosis requires further investigation using dedicated behavioral assays. Therefore, calcium and potassium silicates represent promising tools for integrated management of this invasive pest, potentially reducing chemical pesticide use and promoting sustainable crop protection.

## Conclusion

Calcium and potassium silicate applications significantly enhanced tomato productivity under the conditions of this study, as reflected in increased average fruit weight, higher yield per hectare, and reduced postharvest losses. Both treatments reduced fruit weight loss and decay rates, thereby improving storability and postharvest quality. In addition to improving plant performance, these silicate treatments suppressed *Tuta absoluta* population growth and reproductive potential. Life table analysis indicated a considerable reduction in pest population development relative to the control, along with fewer feeding tunnels on leaves. However, the underlying mechanisms of pest suppression should be interpreted with caution, as behavioral and physiological effects were not independently tested. Overall, the results suggest that calcium and potassium silicates may play a dual role in improving tomato yield and quality while contributing to the suppression of *T. absoluta*. Incorporating these treatments into pest management programs could provide a complementary strategy to reduce reliance on chemical insecticides and support more sustainable tomato production systems.

### Limitations

This study was conducted under controlled field conditions at a single location, which may limit the generalizability of the findings to other environments or tomato cultivars. The experiments focused on a 40–60 day period after treatment, and longer-term effects of silicate applications on *T. absoluta* population dynamics and crop yield were not assessed. Additionally, environmental factors such as temperature, humidity, and rainfall were not varied systematically, and direct measurements of silicon accumulation in leaf tissues or related defense enzyme activities were not performed. Furthermore, behavioral assays (choice/no-choice tests) were not conducted, which prevented a clear distinction between antixenosis and antibiosis mechanisms. Future studies could address these limitations to provide more comprehensive insights into the efficacy of silicate treatments.

## Supplementary Information

Below is the link to the electronic supplementary material.

Supplementary Material 1

## Data Availability

The data supporting the findings of this study are available from the corresponding author upon reasonable request.
