# Supplementary material for: Calcium and potassium silicates impact life table parameters of Tuta absoluta (Lepidoptera: Gelechiidae) and improve field performance of tomato (Solanum lycopersicum)
Source: Sci Rep. 2026 May 9;16:22998. doi: 10.1038/s41598-026-51521-y (PMC13392295; doi:10.1038/s41598-026-51521-y)
Supplement: Supplementary file 1 — Supplementary Material 1 [file 41598_2026_51521_MOESM1_ESM.pdf]

COVER LETTER

(Manuscript submission)

Date: 2025-06-14

Dear Chief Editor,

I am enclosing herewith a manuscript entitled **"Calcium and Potassium Silicates Impact Life Table Parameters of *Tuta absoluta* (Lepidoptera: Gelechiidae) and Improve Field Performance of Tomato (*Solanum lycopersicum*)"** For publication in **Scientific Reports** for possible evaluation. The Corresponding author of this manuscript is **Shahram Aramideh** and contribution of the authors as mentioned below with their responsibility in the research.

sh.aramideh@urmia.ac.ir

1. UNDERTAKING

With this manuscript submission, I hereby declare that:

All authors of this article have directly participated in the planning, execution, or analysis of this study;

All authors of this paper have read and approved the final version submitted;

All authors have no conflict of interest associated or affiliated with this publication;

The contents of this manuscript have not been copyrighted or published previously;

The contents of this manuscript are not now under consideration for publication elsewhere.

2. RESEARCH AND MANUSCRIPT RELATED DETAILS

Submitted manuscript is a ***Research Article***

3. GRANTS OR FUNDING INFORMATION

My Research Project was **not** sponsored.

Signature: *Shahram Aramideh*
